# Supplementary material for: Monosaccharides drive Salmonella gut colonization in a context-dependent or -independent manner
Source: Nat Commun. 2025 Feb 18;16:1735. doi: 10.1038/s41467-025-56890-y (PMC11836396; doi:10.1038/s41467-025-56890-y)
Supplement: Supplementary file 1 — Supplementary Information [file 41467_2025_56890_MOESM1_ESM.pdf]

Supplementary Information

Monosaccharides drive *Salmonella* gut colonization in a context-dependent or -independent manner

Christopher Schubert[1*], Bidong D. Nguyen[1], Andreas Sichert[2], Nicolas Näpflin[3], Anna Sintsova[1], Lilith Feer[1], Jana Näf[1], Benjamin B.J. Daniel[1], Yves Steiger[1], Christian von Mering[3], Uwe Sauer[2], Wolf-Dietrich Hardt[1*]

Affiliations:

[1]Institute of Microbiology, Department of Biology, ETH Zurich, Zurich, Switzerland

[2]Institute of Molecular Systems Biology, ETH Zurich, Zurich, Switzerland

[3]Department of Molecular Life Sciences and Swiss Institute of Bioinformatics, University of Zurich, Zurich, Switzerland

*for correspondence: cschubert@ethz.ch and hardt@micro.biol.ethz.ch

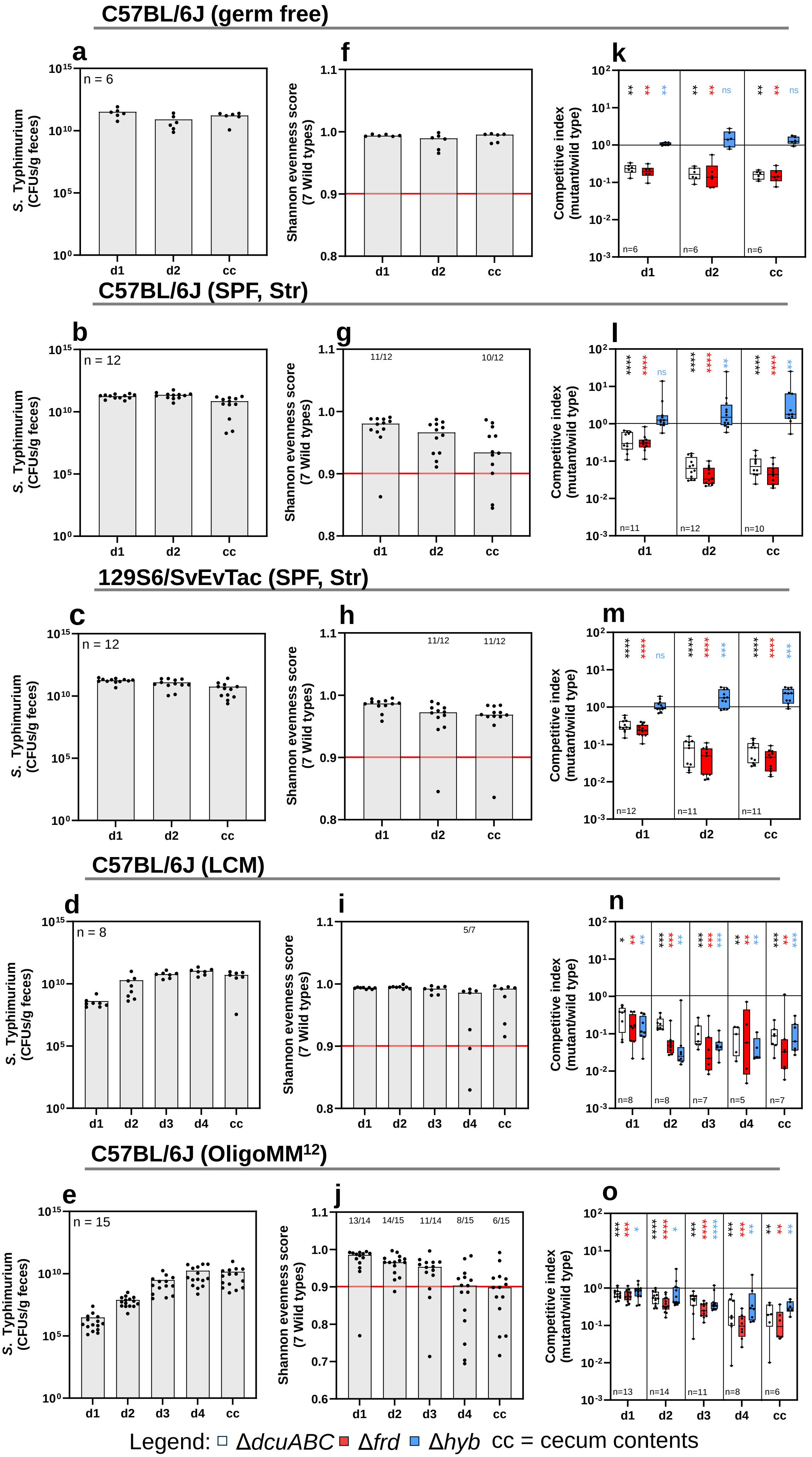

C57BL/6J (germ free)
a
n = 6
S. Typhimurium (CFUs/g feces)
d1
d2
cc
f
Shannon evenness score (7 Wild types)
k
Competitive index (mutant/wild type)
** ** **
** ** ns
** ** ns
n=6
C57BL/6J (SPF, Str)
b
n = 12
g
11/12
10/12
l
**** **** ns
**** **** **
**** **** **
n=11
n=12
n=10
129S6/SvEvTac (SPF, Str)
c
n = 12
h
11/12
11/12
m
**** **** ns
**** **** ***
**** **** ***
n=12
n=11
n=11
C57BL/6J (LCM)
d
n = 8
d3
d4
i
5/7
n
* ** **
*** *** **
*** *** ***
** ** **
*** ** ***
n=8
n=8
n=7
n=5
n=7
C57BL/6J (OligoMM12)
e
n = 15
j
13/14
14/15
11/14
8/15
6/15
o
*** *** *
**** **** *
*** **** ****
*** *** **
** ** **
n=13
n=14
n=11
n=8
n=6
Legend: ΔdcuABC Δfrd Δhyb cc = cecum contents

Supplementary Figure 1. WISH-barcoded *S*. Typhimurium pool provides reproducible data throughout all tested mouse models.

The overview shows the *S*. Typhimurium bacterial loads for the mutant pool, Shannon evenness score of the 7 wild types, and the competitive index of the three control mutants, Δ*dcuABC*, Δ*frd*, and Δ*hyb* in all tested mouse models, as indicated above each section. Panels (**a-e**) are shown as bar plots, displaying the median with all data points. These represent total *S*. Typhimurium colony-forming units (CFUs) per gram of feces or cecum content (cc), with the initial sample size for each mouse model indicated. For panels (**f-j**), the Shannon evenness score (SES) was calculated for the seven WISH-barcoded SL1344 wild types. The red line indicates the SES of 0.9, which served as the cutoff for further analysis. The number above each bar indicates the number of samples within this threshold. Panels (**f–j**) present the data in bar plots, showing the median and individual data points. For panels (**k-o**), the competitive index of the three control mutants in the *S.* Typhimurium mutant pool (Δ*dcuABC*, Δ*frd*, and Δ*hyb*) is shown and was statistically compared to the SL1344 wild type in the control group. The black line indicates a wild-type competitive index of 1. The competitive experiments are presented as box-and-whisker plots, displaying the median, interquartile range (25th to 75th percentiles), minimum and maximum values, and individual data points. *P* values were calculated using the two tailed Mann–Whitney *U*-test: **** ≙ $P < 0.0001$; *** ≙ $P < 0.0005$; ** ≙ $P < 0.005$; * ≙ $P < 0.05$; ns ≙ $P > 0.05$. The source data for panels (**a-o**) are provided in the Source Data file.

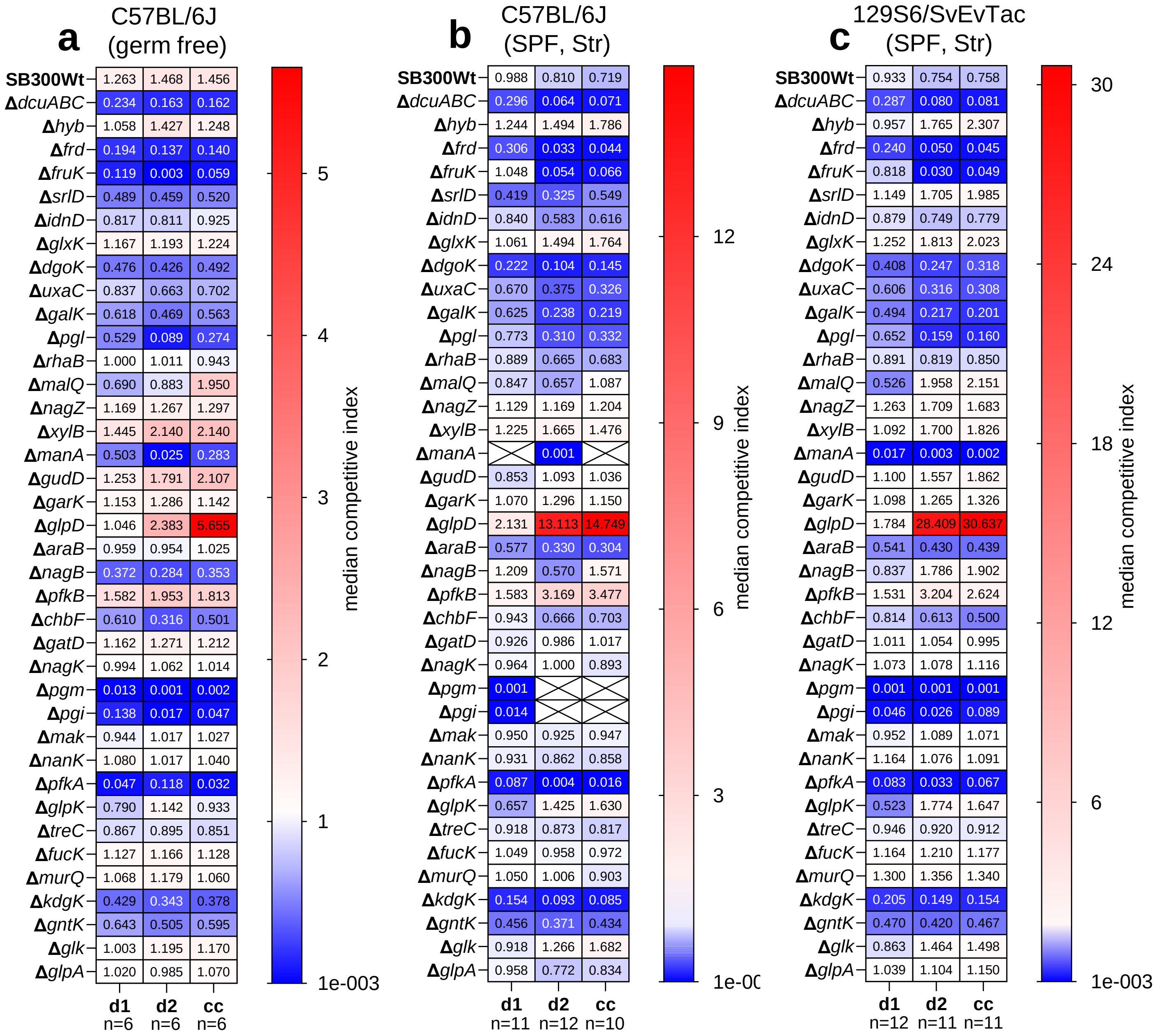

Supplementary Figure 2. Heatmap showing the fitness of WISH-barcoded SL1344 mutants in germ free and perturbed mouse models. For panels (**a-c**), the fitness effects of *S.* Typhimurium mutants defective in carbohydrate utilization are shown as the median competitive index for each gene indicated on the y-axis. The mouse models are listed above each heatmap, and values below 1 indicate a fitness defect compared to the wild-type strain (blue). A crossed-out rectangle indicates that all individual fitness data points are below the limit of detection. The sample size (n) for each day post-infection is indicated on the x-axis. The source data for panels (**a-c**) are provided in the Source Data file.

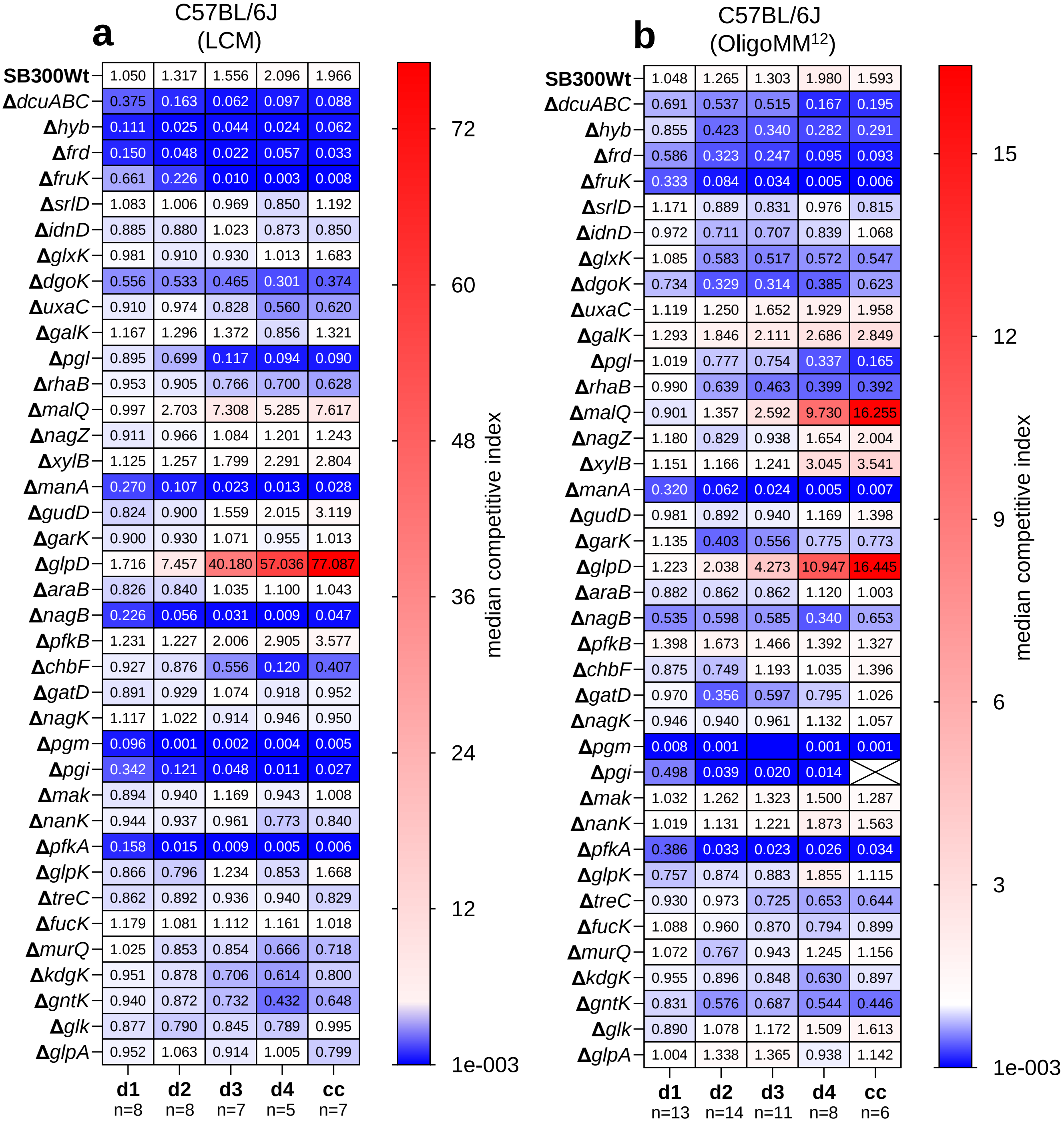

Supplementary Figure 3. Heatmap showing the fitness of WISH-barcoded SL1344 mutants in the gnotobiotic mouse models. For panels (**a, b**), the fitness effects of *S.* Typhimurium mutants defective in carbohydrate utilization are shown as the median competitive index for each gene indicated on the y-axis. The mouse models are listed above each heatmap, and values below 1 indicate a fitness defect compared to the wild-type strain (blue). A crossed-out rectangle indicates that all individual fitness data points are below the limit of detection. The sample size (n) for each day post-infection is indicated on the x-axis. The source data for panels (**a, b**) are provided in the Source Data file.

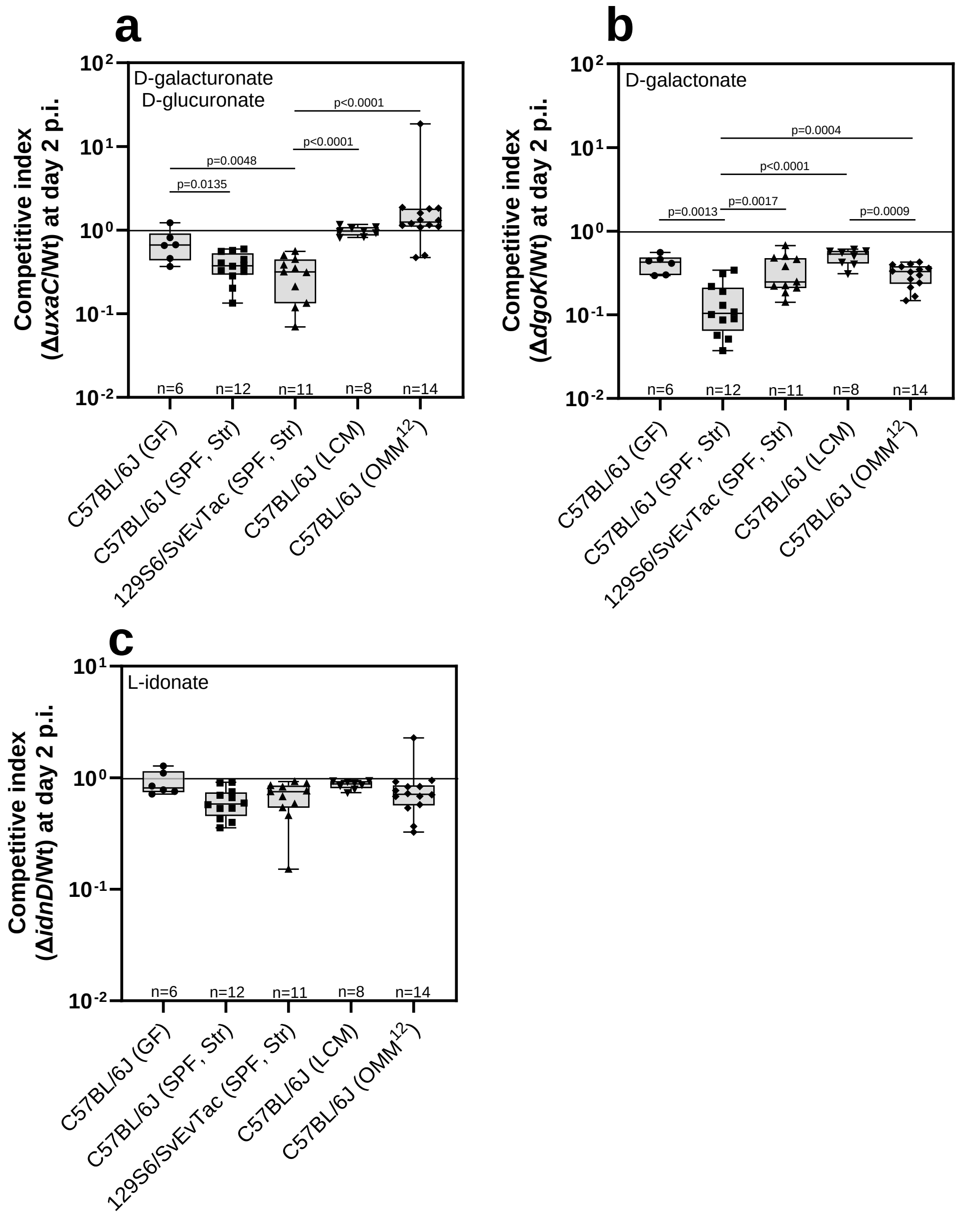

Supplementary Figure 4. D-galacturonate and D-galactonate are utilized in a context-dependent manner, whereas L-idonate is not. For panels (**a–c**), the competitive index 2 days post-infection is plotted for the Δ*dgoK* (D-galactonate), Δ*uxaC* (D-galacturonate), and Δ*idnD* (L-idonate) mutants across all five mouse models, as indicated on the x-axis. Box-and-whisker plots display the median, interquartile range (25th to 75th percentiles), minimum and maximum values, and individual data points. The sample size (n) is indicated on the x-axis, based on data from at least two independent experiments. *P* values were calculated using the two tailed Mann–Whitney *U*-test. The source data for panels (**a-c**) are provided in the Source Data file.

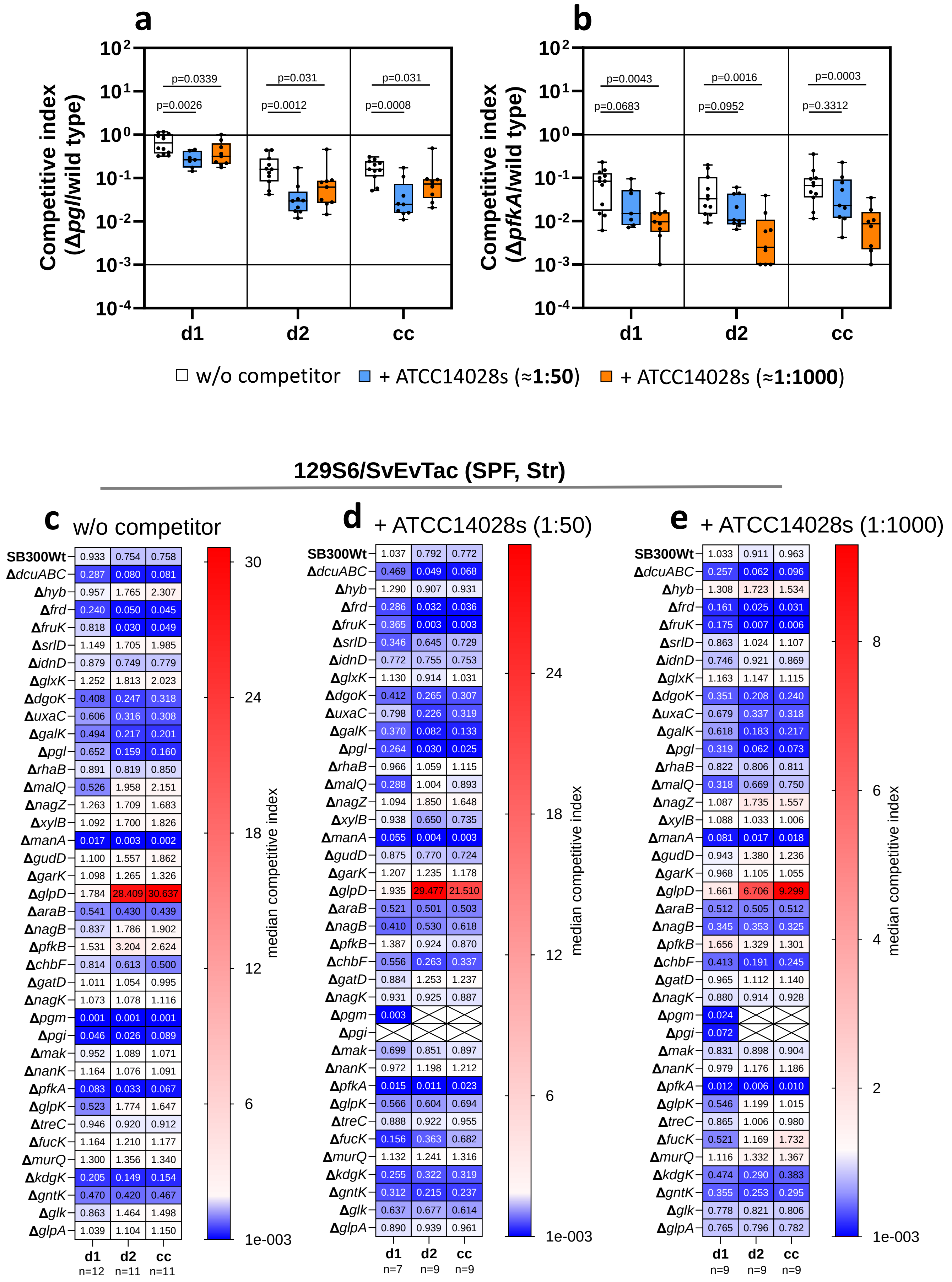
a
Competitive index (Δpgl/wild type)
10^2 10^1 10^0 10^-1 10^-2 10^-3 10^-4
p=0.0339 p=0.0026
p=0.031 p=0.0012
p=0.031 p=0.0008
d1 d2 cc
b
Competitive index (ΔpfkA/wild type)
p=0.0043 p=0.0683
p=0.0016 p=0.0952
p=0.0003 p=0.3312
d1 d2 cc
w/o competitor
+ ATCC14028s (≈1:50)
+ ATCC14028s (≈1:1000)
129S6/SvEvTac (SPF, Str)
c w/o competitor
SB300Wt 0.933 0.754 0.758
ΔdcuABC 0.287 0.080 0.081
Δhyb 0.957 1.765 2.307
Δfrd 0.240 0.050 0.045
ΔfruK 0.818 0.030 0.049
ΔsrlD 1.149 1.705 1.985
ΔidnD 0.879 0.749 0.779
ΔglxK 1.252 1.813 2.023
ΔdgoK 0.408 0.247 0.318
ΔuxaC 0.606 0.316 0.308
ΔgalK 0.494 0.217 0.201
Δpgl 0.652 0.159 0.160
ΔrhaB 0.891 0.819 0.850
ΔmalQ 0.526 1.958 2.151
ΔnagZ 1.263 1.709 1.683
ΔxylB 1.092 1.700 1.826
ΔmanA 0.017 0.003 0.002
ΔgudD 1.100 1.557 1.862
ΔgarK 1.098 1.265 1.326
ΔglpD 1.784 28.409 30.637
ΔaraB 0.541 0.430 0.439
ΔnagB 0.837 1.786 1.902
ΔpfkB 1.531 3.204 2.624
ΔchbF 0.814 0.613 0.500
ΔgatD 1.011 1.054 0.995
ΔnagK 1.073 1.078 1.116
Δpgm 0.001 0.001 0.001
Δpgi 0.046 0.026 0.089
Δmak 0.952 1.089 1.071
ΔnanK 1.164 1.076 1.091
ΔpfkA 0.083 0.033 0.067
ΔglpK 0.523 1.774 1.647
ΔtreC 0.946 0.920 0.912
ΔfucK 1.164 1.210 1.177
ΔmurQ 1.300 1.356 1.340
ΔkdgK 0.205 0.149 0.154
ΔgntK 0.470 0.420 0.467
Δglk 0.863 1.464 1.498
ΔglpA 1.039 1.104 1.150
d1 n=12 d2 n=11 cc n=11
30 24 18 12 6 1e-003
median competitive index
d + ATCC14028s (1:50)
SB300Wt 1.037 0.792 0.772
ΔdcuABC 0.469 0.049 0.068
Δhyb 1.290 0.907 0.931
Δfrd 0.286 0.032 0.036
ΔfruK 0.365 0.003 0.003
ΔsrlD 0.346 0.645 0.729
ΔidnD 0.772 0.755 0.753
ΔglxK 1.130 0.914 1.031
ΔdgoK 0.412 0.265 0.307
ΔuxaC 0.798 0.226 0.319
ΔgalK 0.370 0.082 0.133
Δpgl 0.264 0.030 0.025
ΔrhaB 0.966 1.059 1.115
ΔmalQ 0.288 1.004 0.893
ΔnagZ 1.094 1.850 1.648
ΔxylB 0.938 0.650 0.735
ΔmanA 0.055 0.004 0.003
ΔgudD 0.875 0.770 0.724
ΔgarK 1.207 1.235 1.178
ΔglpD 1.935 29.477 21.510
ΔaraB 0.521 0.501 0.503
ΔnagB 0.410 0.530 0.618
ΔpfkB 1.387 0.924 0.870
ΔchbF 0.556 0.263 0.337
ΔgatD 0.884 1.253 1.237
ΔnagK 0.931 0.925 0.887
Δpgm 0.003
Δpgi
Δmak 0.699 0.851 0.897
ΔnanK 0.972 1.198 1.212
ΔpfkA 0.015 0.011 0.023
ΔglpK 0.566 0.604 0.694
ΔtreC 0.888 0.922 0.955
ΔfucK 0.156 0.363 0.682
ΔmurQ 1.132 1.241 1.316
ΔkdgK 0.255 0.322 0.319
ΔgntK 0.312 0.215 0.237
Δglk 0.637 0.677 0.614
ΔglpA 0.890 0.939 0.961
d1 n=7 d2 n=9 cc n=9
24 18 12 6 1e-003
median competitive index
e + ATCC14028s (1:1000)
SB300Wt 1.033 0.911 0.963
ΔdcuABC 0.257 0.062 0.096
Δhyb 1.308 1.723 1.534
Δfrd 0.161 0.025 0.031
ΔfruK 0.175 0.007 0.006
ΔsrlD 0.863 1.024 1.107
ΔidnD 0.746 0.921 0.869
ΔglxK 1.163 1.147 1.115
ΔdgoK 0.351 0.208 0.240
ΔuxaC 0.679 0.337 0.318
ΔgalK 0.618 0.183 0.217
Δpgl 0.319 0.062 0.073
ΔrhaB 0.822 0.806 0.811
ΔmalQ 0.318 0.669 0.750
ΔnagZ 1.087 1.735 1.557
ΔxylB 1.088 1.033 1.006
ΔmanA 0.081 0.017 0.018
ΔgudD 0.943 1.380 1.236
ΔgarK 0.968 1.105 1.055
ΔglpD 1.661 6.706 9.299
ΔaraB 0.512 0.505 0.512
ΔnagB 0.345 0.353 0.325
ΔpfkB 1.656 1.329 1.301
ΔchbF 0.413 0.191 0.245
ΔgatD 0.965 1.112 1.140
ΔnagK 0.880 0.914 0.928
Δpgm 0.024
Δpgi 0.072
Δmak 0.831 0.898 0.904
ΔnanK 0.979 1.176 1.186
ΔpfkA 0.012 0.006 0.010
ΔglpK 0.546 1.199 1.015
ΔtreC 0.865 1.006 0.980
ΔfucK 0.521 1.169 1.732
ΔmurQ 1.116 1.332 1.367
ΔkdgK 0.474 0.290 0.383
ΔgntK 0.355 0.253 0.295
Δglk 0.778 0.821 0.806
ΔglpA 0.765 0.796 0.782
d1 n=9 d2 n=9 cc n=9
8 6 4 2 1e-003
median competitive index

Supplementary Figure 5: Intraspecies competition between the two *S.* Typhimurium strains, SL1344 and ATCC14028s, is highly specific. Panel (**a, b**) shows the Δ*pgl* and Δ*pfkA* competitive index in the presence of ATCC14028s at 1:50 ratios (n = 7 for day 1; n = 9 for day 2 and cecal content) and 1:1000 ratios (n = 9). It also includes data in the absence of ATCC14028s (day 1: n = 12; day 2 and cecal content: n = 11). *pgl* encodes the enzyme for the second step of the oxidative pentose phosphate pathway, while *pfkA* encodes phosphofructokinase, a key enzyme in glycolysis. Box-and-whisker plots display the median, interquartile range (25th to 75th percentiles), minimum and maximum values, and individual data points. Panels (**c-e**) display the remaining fitness effects of *S*. Typhimurium mutants defective in carbohydrate utilization as a heatmap, with each gene indicated on the y-axis. Fitness is assessed without an additional competitor, with ATCC14028s at a 1:50 ratio and at a 1:1000 ratio, as noted above each respective heatmap. The sample size (n) is indicated below the x-axis. A crossed-out rectangle indicates that all individual competitive index data points are below the limit of detection. Values below 1 indicate a fitness defect compared to the wild-type strain (blue). *P* values were calculated using the two tailed Mann–Whitney *U*-test. The source data for panels (**a-e**) are provided in the Source Data file.

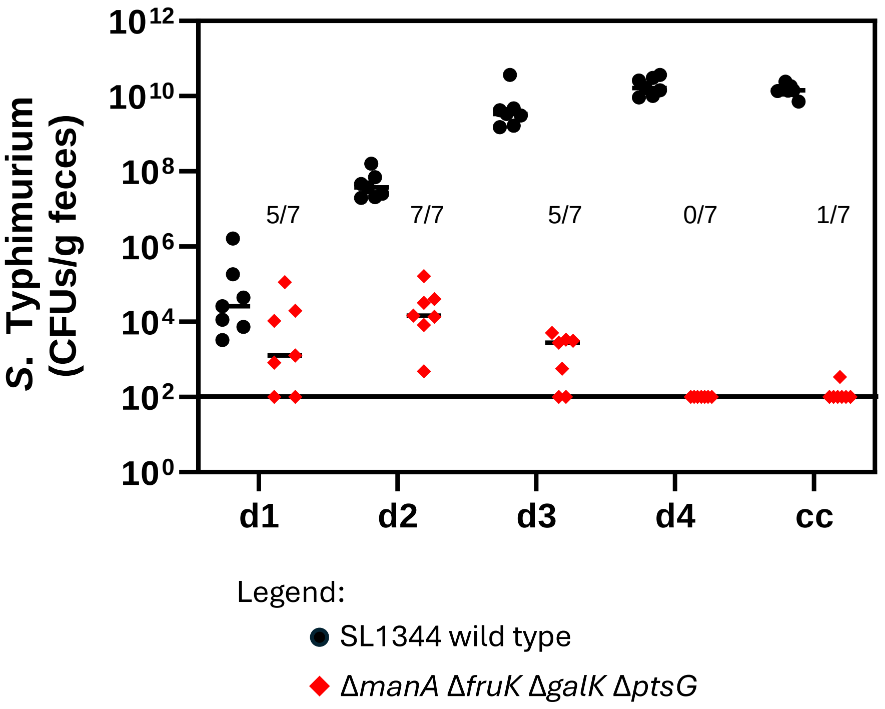

Supplementary Figure 6: The Δ*manA* Δ*fruK* Δ*galK* Δ*ptsG* mutant is displaced by the wild type in OligoMM^{12} mice. Competitive infection of C57BL/6J mice associated with the OligoMM^{12} microbiota using SL1344 wild type (circle; n = 7) and Δ*manA* Δ*fruK* Δ*galK* Δ*ptsG* quadruple mutant (red diamond; n = 7, from at least two independent experiments). The bacterial loads are plotted in colony-forming units (CFUs) per gram of feces or cecum content (cc) for each day post-infection. The detection limit is indicated by a black line, and the numbers above show how many samples are above this limit. The source data for the panel is provided in the Source Data file.

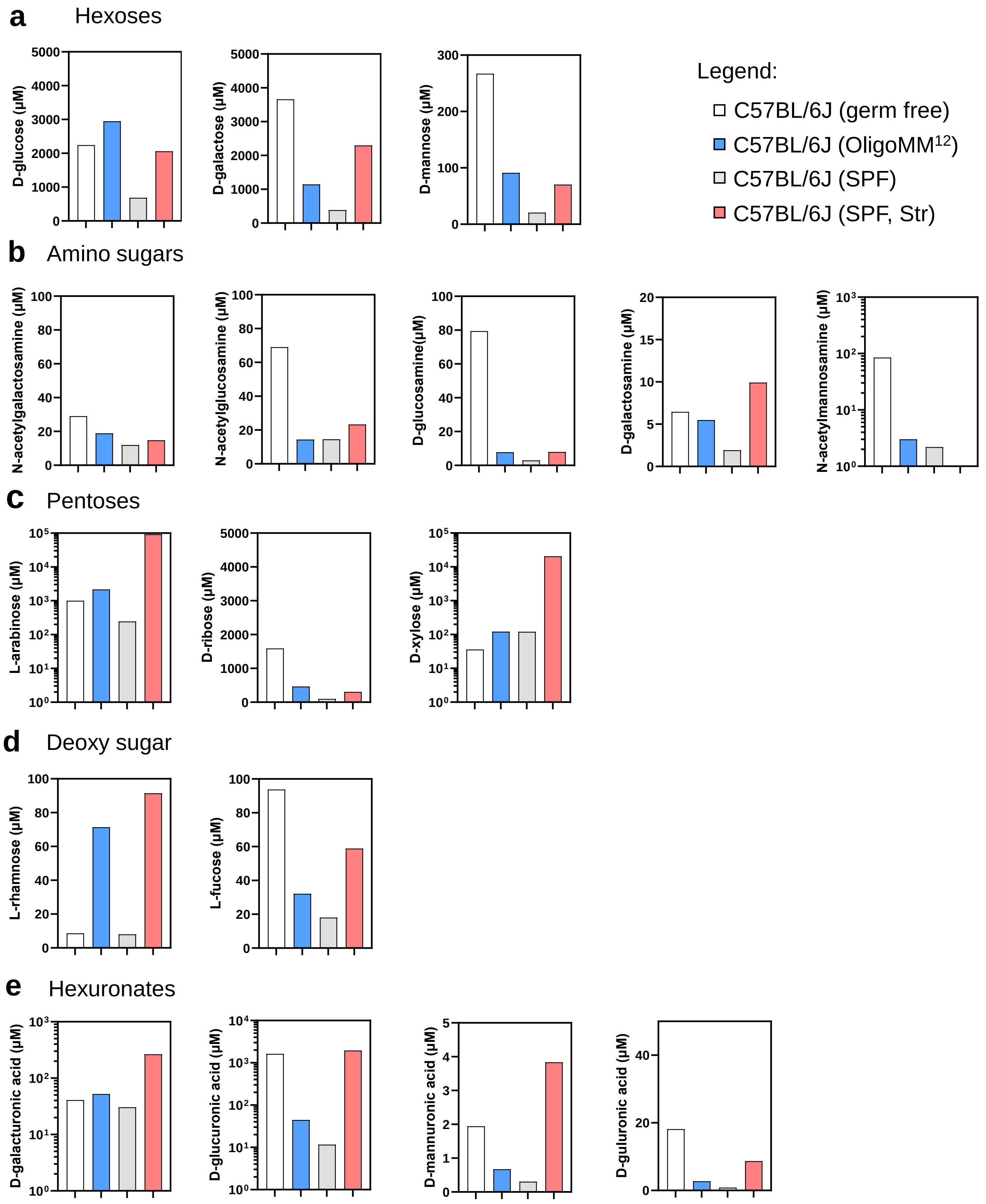

a Hexoses
D-glucose (μM)
D-galactose (μM)
D-mannose (μM)
Legend:
C57BL/6J (germ free)
C57BL/6J (OligoMM12)
C57BL/6J (SPF)
C57BL/6J (SPF, Str)
b Amino sugars
N-acetylgalactosamine (μM)
N-acetylglucosamine (μM)
D-glucosamine(μM)
D-galactosamine (μM)
N-acetylmannosamine (μM)
c Pentoses
L-arabinose (μM)
D-ribose (μM)
D-xylose (μM)
d Deoxy sugar
L-rhamnose (μM)
L-fucose (μM)
e Hexuronates
D-galacturonic acid (μM)
D-glucuronic acid (μM)
D-mannuronic acid (μM)
D-guluronic acid (μM)

Supplementary Figure 7: Quantification of free monosaccharides by LC-MS in cecum content of germ-free, OligoMM^{12}-associated, unperturbed, and streptomycin pretreated SPF C57BL/6J mice. The absolute measurements of free monosaccharides in cecum contents are presented in separate plots, showing median values on a linear y-axis scale. Due to variability within the data, N-acetylmannosamine, L-arabinose, D-xylose, D-galacturonic acid, and D-glucuronic acid measurements are presented on a logarithmic scale for clarity. Data from germ-free C57BL/6J (n = 5; white) and OligoMM^{12} models (n = 5; blue) were compared to a previously published dataset of SPF C57BL/6J mice (n = 5; grey) and streptomycin pretreated SPF C57BL/6J mice (n = 5; red)[1]. For panels (**a–e**), the monosaccharide groups are indicated above each subpanel section. The source data for panels (**a-e**) are provided in the Source Data file.

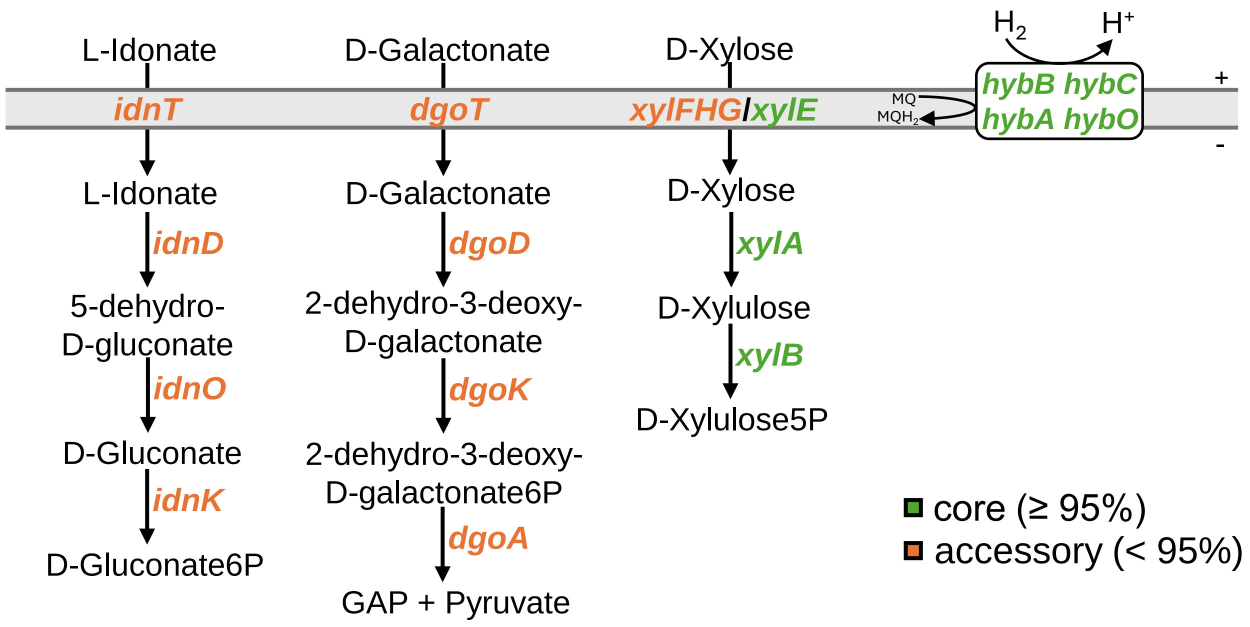

Supplementary Figure 8: Analysis of gene presence for context-dependent carbohydrate utilization. The genomes of non-typhoidal *Salmonella*, *Citrobacter*, *Shigella*, and *Escherichia* were analyzed for the presence of genes involved in the degradation of L-idonate (*idn* operon), D-galactonate (*dgo* operon), D-xylose (*xyl* operon), and hydrogen (*hyb* operon). A schematic representation of the L-idonate, D-galactonate, D-xylose, and hydrogen utilization pathways is shown, with each enzymatic step indicated. If a gene is present in at least 95% of all analyzed genomes, it is termed core (green). Less than 95%, it is termed accessory (orange). The full names of the abbreviations are listed in Supplementary Table S12. The source data for the panel are provided in the Source Data file.

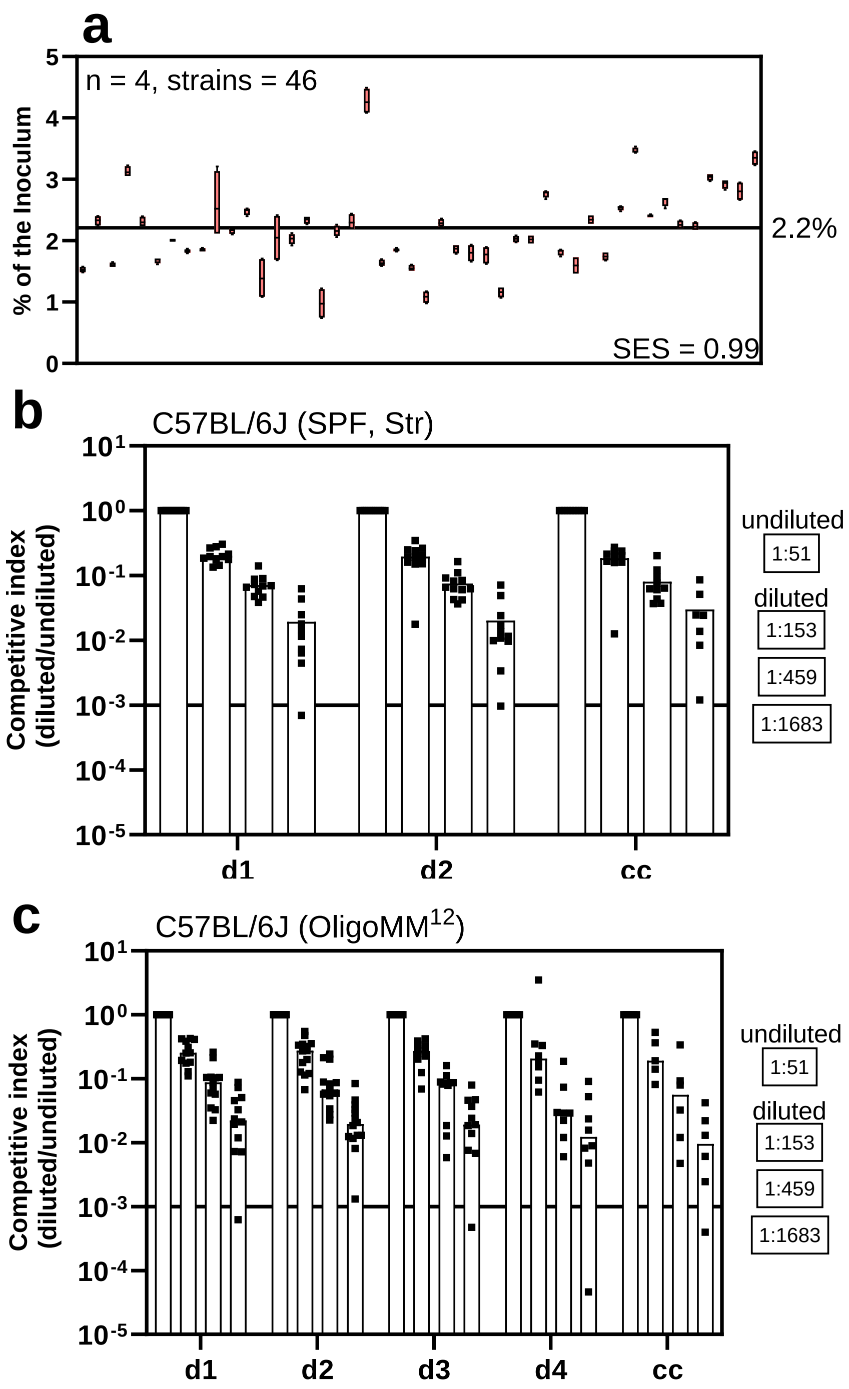
a
n = 4, strains = 46
% of the Inoculum
2.2%
SES = 0.99
b
C57BL/6J (SPF, Str)
Competitive index (diluted/undiluted)
d1
d2
cc
undiluted
1:51
diluted
1:153
1:459
1:1683
c
C57BL/6J (OligoMM12)
Competitive index (diluted/undiluted)
d1
d2
d3
d4
cc
undiluted
1:51
diluted
1:153
1:459
1:1683

Supplementary Figure 9: Individual strain distribution in the inoculum and the limit of detection in streptomycin-pretreated SPF C57BL/6J mice and gnotobiotic OligoMM^{12} mice. a The Shannon evenness score (SES) was calculated for all 46 SL1344 strains in the pool, excluding the wild-type titration standards. The y-axis indicates the proportion of each WISH-barcoded SL1344 strain within the inoculum (n = 4) after 4 h of enrichment at 37°C. The average abundance across all mutants in the inoculum is approximately 2.2%, as indicated in the diagram. The SL1344 mutant pool achieves an SES of 0.99. Box-and-whisker plots display the median, interquartile range (25th to 75th percentiles), minimum and maximum values, and individual data points. Panels (**b, c**) display the competitive index of the titration standards, normalized to an undiluted SL1344 wild type, for the C57BL/6J (SPF, Str) mouse model (day 1: n = 11; day 2: n = 12; cecal content: n = 10) and the C57BL/6J (OligoMM^{12}) mouse model (day 1: n = 13; day 2: n = 14; day 3: n = 11; day 4: n = 8; cecal content: n = 6). Box-and-whisker plots (**b, c**) display the median, interquartile range (25th to 75th percentiles), minimum and maximum values, and individual data points. The dilution ratios are indicated on the right side of the diagram. We still have excellent resolution at the lowest titration level. For this reason, we conservatively set the detection limit to 10^{-3}; however, this limit is directly correlated with sequencing depth. The source data for panels (**a-c**) are provided in the Source Data file.

References:

1. Nguyen, B.D. et al. *Salmonella* Typhimurium screen identifies shifts in mixed-acid fermentation during gut colonization. Cell Host Microbe (2024).
