# Supplementary material for: Monosaccharides drive Salmonella gut colonization in a context-dependent or -independent manner
Source: Nat Commun. 2025 Feb 18;16:1735. doi: 10.1038/s41467-025-56890-y (PMC11836396; doi:10.1038/s41467-025-56890-y)
Supplement: Supplementary file 2 — Description of Additional Supplementary Files [file 41467_2025_56890_MOESM2_ESM.pdf]

Monosaccharides drive *Salmonella* gut colonization in a context-dependent or -independent manner

Christopher Schubert[1*], Bidong D. Nguyen[1], Andreas Sichert[2], Nicolas Näpflin[3], Anna Sintsova[1], Lilith Feer[1], Jana Näf[1], Benjamin B.J. Daniel[1], Yves Steiger[1], Christian von Mering[3], Uwe Sauer[2], Wolf-Dietrich Hardt[1*]

Affiliations:

[1]Institute of Microbiology, Department of Biology, ETH Zurich, Zurich, Switzerland

[2]Institute of Molecular Systems Biology, ETH Zurich, Zurich, Switzerland

[3]Department of Molecular Life Sciences and Swiss Institute of Bioinformatics, University of Zurich, Zurich, Switzerland

*for correspondence: cschubert@ethz.ch and hardt@micro.biol.ethz.ch

Description of additional Supplementary Data

Supplementary Data 1: Overview of the WISH-barcoded *S.* Typhimurium pool, including controls, carbohydrate mutants, and WISH-tag sequences.

Supplementary Data 2: Raw WISH-barcode counts from the C57BL/6J (germ-free) model, including Shannon evenness scores and competitive index calculations.

Supplementary Data 2.1: Competitive index (CI) calculations for individual C57BL/6J (germ-free) mice, along with Shannon evenness scores (SES) indicating data excluded from further analysis due to SES scores below 0.9. Also includes the median, minimum, and maximum CI values for each strain per day post infection.

Supplementary Data 3: Raw WISH-barcode counts from the streptomycin pretreated C57BL/6J (SPF, Str) model, including Shannon evenness scores and competitive index calculations.

Supplementary Data 3.1: Competitive index (CI) calculations for individual streptomycin pretreated C57BL/6J (SPF, Str) mice, along with Shannon evenness scores (SES) indicating data excluded from further analysis due to SES scores below 0.9. Also includes the median, minimum, and maximum CI values for each strain per day post infection.

Supplementary Data 4: Raw WISH-barcode counts from the streptomycin pretreated 129S6/SvEvTac (SPF, Str) model, including Shannon evenness scores and competitive index calculations.

Supplementary Data 4.1: Competitive index (CI) calculations for individual streptomycin pretreated 129S6/SvEvTac (SPF, Str) mice, along with Shannon evenness scores (SES) indicating data excluded from further analysis due to SES scores below 0.9. Also includes the median, minimum, and maximum CI values for each strain per day post infection.

Supplementary Data 5: Raw WISH-barcode counts from the C57BL/6J (LCM) model, including Shannon evenness scores and competitive index calculations.

Supplementary Data 5.1: Competitive index (CI) calculations for individual C57BL/6J (LCM) mice, along with Shannon evenness scores (SES) indicating data excluded from further

analysis due to SES scores below 0.9. Also includes the median, minimum, and maximum CI values for each strain per day post infection.

Supplementary Data 6: Raw WISH-barcode counts from the C57BL/6J (OligoMM^{12}) model, including Shannon evenness scores and competitive index calculations.

Supplementary Data 6.1: Competitive index (CI) calculations for individual C57BL/6J (OligoMM^{12}) mice, along with Shannon evenness scores (SES) indicating data excluded from further analysis due to SES scores below 0.9. Also includes the median, minimum, and maximum CI values for each strain per day post infection.

Supplementary Data 7: Raw WISH-barcode counts from the streptomycin pretreated 129S6/SvEvTac (SPF, Str) model for the competition experiments with ATCC14028s, including Shannon evenness scores and competitive index calculations.

Supplementary Data 7.1: Competitive index (CI) calculations for individual streptomycin pretreated 129S6/SvEvTac (SPF, Str) mice for the competition experiments with ATCC14028s, along with Shannon evenness scores (SES) indicating data excluded from further analysis due to SES scores below 0.9. Also includes the median, minimum, and maximum CI values for each strain per day post infection.

Supplementary Data 8: Bioinformatic analysis of metabolic gene presence in *Salmonella*, *Escherichia*, *Shigella*, and *Citrobacter*.

Supplementary Data 8.1: Accession numbers of genomes selected for the bioinformatic analysis.

Supplementary Data 9: Bacterial strains used in this study.

Supplementary Data 10: Plasmids used in this study.

Supplementary Data 11: Oligonucleotides used in this study.

Supplementary Data 12: Abbreviations used in Fig. 1d, Fig. 5b, and Supplementary Fig. S8.
